# Destruction/Inactivation of SARS-CoV-2 Virus Using Ultrasound Excitation: A Preliminary Study

**DOI:** 10.3390/v18020152

**Published:** 2026-01-23

**Authors:** Almunther Alhasawi, Fajer Alassaf, Alshimaa Hassan

**Affiliations:** 1 Infectious Diseases Hospital, Ministry of Health, Kuwait; awalhasawi@moh.gov.kw; 2 Virology Lab-Farwaniya Hospital, Ministry of Health, Kuwait; drfajer@gmail.com

**Keywords:** SARS-CoV-2, ultrasound waves, viral load reduction, cycle threshold (Ct) values, enveloped viruses, mechanical disruption, therapeutic strategy, experimental study

## Abstract

SARS-CoV-2, the causative virus of the COVID-19 pandemic, is a highly transmissible, enveloped, single-stranded RNA virus that has mutated into several variants, complicating vaccine strategies and drug resistance. Novel treatment modalities targeting conserved structural vulnerable points are essential to combat these variants. The primary aim of the current study is to test the mechanical vulnerability of the SARS-CoV-2 virus envelope and spike proteins to focused, high-frequency ultrasound waves (25 MHz) in vitro. Utilizing a preliminary pretest and posttest study design, the study was conducted on a virus sample within a distilled water matrix, under controlled laboratory biosafety conditions. Since detailed imaging tools were unavailable, viral disruption was indirectly measured using real-time PCR cycle threshold (Ct) values. Ct values increased significantly after high-frequency ultrasound exposure, indicating a reduction in amplifiable viral genomic material. A paired t-test indicated a significant difference between the pretest and posttest Ct (*p* < 0.001), which is supported by Monte Carlo test results that revealed statistically significant shifting in viral load categories (*p* = 0.001, two-sided). Specifically, 85.7% of high-viral-load samples converted to low or moderate content, 46.7% of low or moderate samples were shifted to negative content. This intervention produced a large effect size (Cohen’s d = 2.422). These results indicate that ultrasound may offer a promising non-pharmacological approach to destroy or inactivate SARS-CoV-2 variants in an aqueous environment.

## 1. Introduction

Viral outbreaks have caused severe global morbidity and mortality over the past century. Five hundred million people were infected, and at least 500 million deaths were attributed to influenza in the 1918 influenza pandemic [1]. Human immunodeficiency virus (HIV) has been responsible for about 44.1 million deaths, while approximately 41 million people were living with it by 2024 [2]. Additionally, hepatitis B virus (HBV) impacts over 250 million people worldwide and led to about 1.1 million deaths in 2022 [3]. The past 20 years have shown high rates of virus-related infections such as the SARS-CoV epidemic in 2003, the swine flu epidemic in 2009, the MERS-CoV epidemic in 2012, the Ebola outbreak in West Africa from 2013 to 2016, the Zika virus disease epidemic from 2015 to 2016, the COVID-19 pandemic spanning from 2019 to 2022, as well as more recent outbreaks like Mpox (2022–2023) and dengue (2024–2025) [2]. Growing global travel, urbanization, and climate change now allow viruses to spread rapidly, increasing the urgency for novel antiviral strategies [4].

As of 1 August 2025, 39 antiviral combinations and 98 antiviral agents have been approved against 13 human viruses, including varicella-zoster virus, cytomegalovirus, herpes simplex virus, HIV, HBV, hepatitis C virus, human papillomavirus, Ebola virus, variola virus, molluscum contagiosum virus, respiratory syncytial virus, SARS-CoV-2, and influenza virus [2]. Most of these agents only suppress replication and do not cure latent or chronic infection [5,6,7,8,9]. Thus, developing low-cost non-pharmacological modalities that can destroy viruses is an ongoing worldwide health priority. Recent advances in physical virology demonstrate that viral particles act as nanoscale mechanical systems with quantifiable stiffness, elasticity, and vibrational modes that can provide such an approach [10,11].

Based on theoretical and computational models, there are specific resonance frequencies within viruses in which externally applied acoustic energy increases oscillation in the capsids, envelopes, or surface glycoproteins, resulting in structural collapse, loss of infectivity, and irreversible deformation [12,13]. Enveloped viruses may also be more prone to mechanical damage in this manner because of their composite structures, which involve a lipid bilayer envelope studded with spike glycoproteins surrounding a protein capsid and nucleic acid core. These spike proteins protrude from the virus surface, indicating mechanically vulnerable and susceptible structures [12]. Ultrasound, an established technology for diagnostic examination and treatment, has thus been recommended as a potential method for the provision of a controlled mechanical energy capable of disrupting the viral ultrastructure without the use of chemical materials [14].

The aim of the current study is to test the mechanical vulnerability of the SARS-CoV-2 virus envelope and spike proteins to focused, high-frequency ultrasound waves in vitro. Since advanced imaging instruments like Atomic Force Microscopy (AFM) or electron microscopy (EM) were not available for biological samples in the study setting, direct visualization of topographical destruction could not be performed. The experimental design was thus adjusted to monitor changes in the Ct values as an indirect measure of viral disruption. Such findings provide preliminary experimental support (Step A) for ultrasound-mediated viral inactivation or structural disruption (indirectly measured via genomic inactivation) and warrant a sequential future validation process, including testing in biological matrices (e.g., blood) and animal models, and by employing high-resolution imaging modalities such as AFM or cryo-EM to correlate acoustic exposure with structural damage. Beyond SARS-CoV-2, if resonance-based mechanical disruption is confirmed and validated by such future studies, it will offer a widely applicable, non-pharmacological strategy against other enveloped viruses that impact current and future pandemics.

## 2. Materials and Methods

### 2.1. Study Design and Setting

A preliminary pretest and post-test study design was used for evaluating the ultrasound effects on SARS-CoV-2. The experiment was conducted in a public health main laboratory in a controlled laboratory environment, equipped with biosafety measures to handle viral samples safely and effectively.

### 2.2. Sample Preparation and Study Experiment

De-identified nasopharyngeal samples were retrospectively obtained from the central virology laboratory repository (stored at 4 °C). Only samples stored for less than 2 months prior to the study were included. The diagnostic Ct values served as the pretest baseline after being adjusted for the dilution factor (0.5 mL VTM:2.5 mL water) to ensure an accurate baseline comparison for the post-test results using the following formula: [∆ Ct = log_2_ (DF)]. To ensure internal validity, sham control (n = 10, Influenza A and B) was performed using the same distilled water, dilution, experimental duration (5 min), and transducer placement, but without ultrasound exposure. No significant Ct changes were observed (*p* > 0.05), which excludes potential environmental degradation or nuclease activity. The experiment was conducted at 22 °C. To maintain genomic stability, all samples were maintained on ice throughout the experiment and processed individually. Following treatment, a same-day posttest RT-PCR assay was performed for all samples while maintaining the cold-chain process. Continuous sample temperature monitoring was not carried out, as the 0.1% duty cycle ensured a non-thermal, mechanical interaction.

A volume of 0.5 mL of viral suspension was added to 2.5 mL of distilled water and mixed well in a 6 mL test tube. An ultrasound transducer was placed inside the tube, maintaining a 2 cm distance from the tip of the transducer to the bottom of the tube to ensure that the virus suspension could be set at the focus length of the transducer.

The ultrasound functioning generator, Textronix AFG31051 (Tektronix, Inc., Beaverton, OR, USA), was configured with the following settings:

- Mode: Burst;
- Frequency: 25 MHz;
- Phase: 0°;
- Amplitude: 7 Vpp;
- Offset: 0 mV;
- Burst Mode Type: N Cycles;
- Cycles per Burst: 25;
- Trigger Delay: 0 ns;
- Trigger Source: internal;
- Trigger Interval: 1 ms (1000 bursts/s);
- Burst Duration: 1 µs (from 25 cycles @ 25 MHz);
- Repetition Rate: 1000 bursts/sec (1 kHz);
- Output Control: Manual (ON/OFF).

The ultrasound intervention was performed at 25 MHz using a 1 µs burst duration and a 1 ms trigger interval (0.1% duty cycle) to ensure a purely mechanical, non-thermal interaction at a calculated acoustic pressure of 107.5 kPa. The power output of the generator was calculated at 7 Vpp to be approximately 122 mW, which is within the 125 mW limit specified for the transducer. Using a 25 MHz focused immersion transducer with a surface area of 3.17 ×10^−5^ m^2^ and a medium approximated to water (density (ρ) = 1000  kg/m^3^), the acoustic pressure was calculated using this equation √2ρcI. The resulting acoustic pressure was 107.5 kPa, slightly exceeding the 0.1 MP threshold needed for virus destruction as suggested by modeling research.

Each sample was exposed to ultrasound for a fixed duration of 5 min. Post ultrasound application, Ct values were estimated again to assess changes in genomic copies.

Viral RNA was extracted from clinical specimens using the KingFisher™ Flex Purification System, using Thermo Scientific™ BindIt™ Software v4.1 (Thermo Fisher Scientific, Waltham, MA, USA) with a magnetic bead-based automated extraction workflow, employing the MagMAX™ Viral/Pathogen II Nucleic Acid Isolation Kit (Thermo Fisher Scientific Baltics UAB, Vilnius, Lithuania) according to the manufacturer’s instructions. Real-time reverse transcription PCR (RT-PCR) was performed using the GeneProof SARS-CoV-2 PCR Kit (GeneProof a.s., Brno, Czech Republic), which targets the E (envelope) and RdRp (RNA-dependent RNA polymerase) genes of SARS-CoV-2, and includes an internal control to monitor extraction efficiency and PCR inhibition. Amplification and fluorescence detection were carried out on the QuantStudio™ Design and Analysis Software v1.6 (Applied Biosystems™, Thermo Fisher Scientific). Thermal cycling conditions were applied as specified by the manufacturer, including a reverse transcription step at 50 °C for 10–15 min, followed by initial denaturation at 95 °C for 2 min, 45 cycles of denaturation at 95 °C for 5–10 s, and a combined annealing/extension step at 60 °C for approximately 40 s, during which fluorescence data were acquired. The following cutoff values were used to categorize the values into groups:

- High Viral Load: Ct < 25;
- Low/moderate Viral Load: Ct 25–38;
- Negative: Ct > 38.

### 2.3. Statistical Analysis

SPSS software Version 27 on the Windows operating system was used to describe and analyze the data. Descriptive statistics, including frequency, mean, and standard deviation of all variables, were performed to present demographic data and Ct values differences. Moreover, the paired-sample *t*-test and Monte Carlo test were used to evaluate the significance of the difference between the pretest and posttest values and viral loads. Effect size was calculated to measure the magnitude of this difference. Cohen’s *d* was calculated using the formula:

$$d=\;\frac{\mathrm{mean}\;\mathrm{difference}}{\;{SD}_{\mathrm{pooled}}},\;{SD}_{\mathrm{pooled}}=\;\sqrt{\frac{{SD}_{\mathrm{pretest}\;}^{2}+{SD}_{\mathrm{posttestt}}^{2}}{2}}$$

## 3. Results

### 3.1. Characteristics of the Study Population and Ct Value Distribution

**Figure 1:**
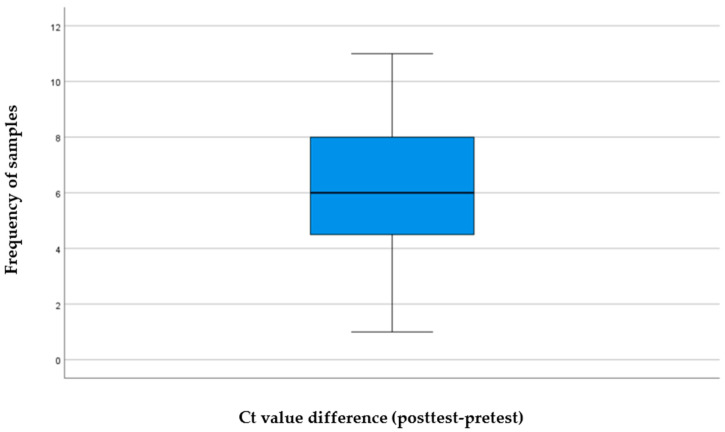
Boxplot of the computed posttest and pretest Ct value differences.

**Figure 2:**
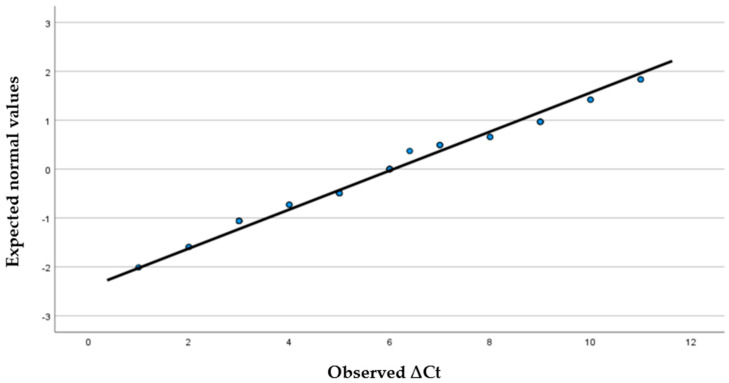
Normal Q-Q plot of the computed posttest and pretest Ct values differences.

Table 1 shows that the age ranged between 1 month and 88 years, with a mean value of 28.67 ± 26.240. Among the study population, 29.5% were aged less than 5 years, 4.5% were aged 5 to 14 years, and 9.1% were aged over 75 years. Regarding gender prevalence, male sex accounted for 40.9% and female sex for 59.1%. Figure 1 provides a boxplot depicting the comparison between the posttest and pretest Ct values, which demonstrates a symmetrical, centered distribution with a value of 6, which is also at the center between the lower third and upper third quartiles. Furthermore, the normal Q-Q plot for the difference scores (Figure 2) suggests that most cases fall approximately on the diagonal reference line, indicating a normal distribution. These findings are also supported by a Shapiro–Wilk test (*p* = 0.126), and hence, the parametric statistical analysis (paired *t*-test) could be used in subsequent analyses.

### 3.2. Changes in Viral Load Categories

It was found that 85.7% of patients with a high viral load in the pretest shifted to low/moderate viral load in the posttest. Additionally, 46.7% of patients with low/moderate viral load became negative in the posttest. The Monte Carlo test results indicated a statistically significant relationship between pre and posttest viral load categories, with a 2-sided *p*-value of 0.001 (99% confidence interval).

**Figure 3:**
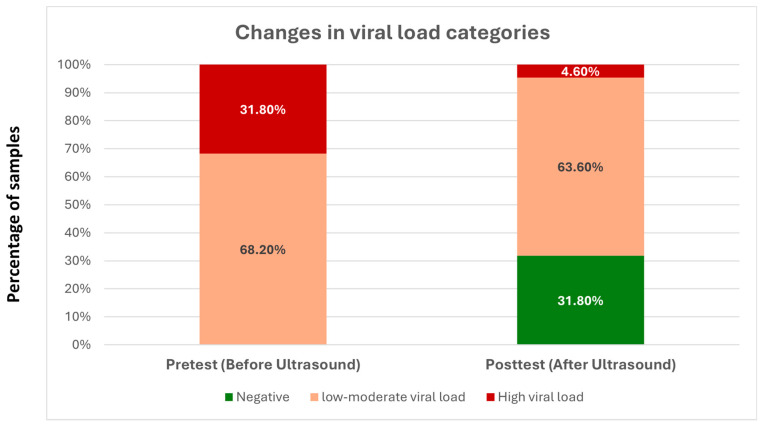
Changes in viral load categories before and after ultrasound exposure.

Table 2 and Figure 3 illustrates the distribution of SARS-CoV-2 real-time RT-PCR cycle threshold (Ct) categories before and after ultrasound exposure. Prior to ultrasound application, 68.2% of samples fell into the low-to-moderate viral load category and 31.8% into the high-viral-load category. Following ultrasound treatment, 31.8% of the samples became PCR-negative, 4.6% remained in the high-viral-load category, and 63.6% were classified as low-to-moderate viral load, indicating a marked shift toward reduced detectable viral RNA after ultrasound exposure.

### 3.3. Paired-Sample t-Test Results

According to Table 3, there was a significant statistical increase in the Ct values from the pretest (mean ± SD = 28.13 ± 7.090) to the post-test (mean ± SD = 34.21 ± 5.908). The *t* value of the mean difference was −16.065 (*p* < 0.001). In addition, the effect size (Cohen’s d) was −2.422.

## 4. Discussion

The current findings indicate that the Ct value of a liquid sample infected with SARS-CoV-2 treated with focused high-frequency ultrasound at 25 MHz has increased significantly, which suggests a decrease in amplifiable viral RNA. The degree of this effect was significant, with a large effect size, indicating that the changes were not only statistically significant but also biologically relevant. These results support the main research hypothesis and suggest that optimal coupling of high-frequency ultrasound to a liquid environment might hinder the viral integrity and replication ability, most probably via mechanical, rather than thermal or chemical mechanisms. The generated frequency-dependent focused experiment tool is promoted by the theoretical and computational models of Wierzbicki et al. [12], who concluded that SARS-CoV-2 displays mechanical resonance properties because of its viscoelastic core–shell structure, including lipid envelope and spike protein assemblies. According to such models, if high acoustic pressure is supplied in a liquid medium, resonance modes are expected to induce envelope instability and spike failure within a few minutes. Importantly, the current study setup met the physical requirements necessary to test this hypothesis: The transducer was directly immersed in the liquid viral medium to eliminate air–liquid impedance mismatch. This ensures true ultrasound propagation through the sample with minimal loss. The acoustic pressure was also determined (~107.5 kPa) as a function of intensity, medium properties, and transducer area. This promotes mechanistic interpretation (e.g., comparison with resonance thresholds predicted by Wierzbicki et al. [12] modeling). The probe concentrates energy at a defined location (focus) in the sample. This creates locally high mechanical stress, unlike diffuse fields created by diagnostic ultrasound probes. Collectively, these conditions facilitate the effective transfer of mechanical energy to viral particles and inform a mechanistic explanation for the observed decrease in viral RNA load.

Recent experimental studies also support the idea that ultrasound can negatively influence the viability of SARS-CoV-2 in vitro, despite the different experimental setups and with different frequency domains. Veras et al. used commercial diagnostic ultrasound probes with different frequencies—3–12, 5–10, and 6–18 MHz—that were used to expose viral suspensions or infected culture media (applied externally through the container wall) for 30 min. The infectivity of the virus was determined by TCID_50_ titer, defined as the median dose required to produce a cytopathic effect in 50% of inoculated cell cultures [15]. Using this measure alongside immunolabelling for spike protein and dsRNA, demonstrating a frequency-dependent decrease in infectivity within the 5–10 MHz band that had reliable efficacy when comparing Wuhan, Delta, and Gamma strains. Critically, though, the researchers maintained the mechanical index within clinical safety limits (~0.3–0.5) without a noticeable increase in temperature, consistent with a mechanical versus thermal influence. High-resolution SEM and AFM imaging further observed aberrant viral morphology, e.g., envelope and spike disruption [16]. Despite the convergent conclusions, the methodological and physical contrasts between the two studies appear substantial and potentially justify the differences in effective frequency ranges.

Medical probes radiate short, broadband acoustic pulses that have multiple harmonic and sideband components, not discrete frequencies [17], and viral suspensions are commonly exposed to these diffuse acoustic fields over long periods (≈30 min in Veras et al.’s study) [2]. Such long exposures may account for cumulative mechanical fatigue and indirect excitation of vulnerable vibrational modes, even when the nominal center frequency is in the low-MHz range. In comparison, the current study utilized a narrowband focused immersion transducer at a specific frequency (25 MHz) directly coupled with the viral medium and delivered for only 5 min, yet it achieved a significant increase in Ct values and a large effect size. This means that efficient acoustic coupling, frequency specificity, and focal pressure concentration could offset shorter exposure times by providing mechanical energy to the viral particles directly. Therefore, variations in effective frequencies and durations of exposure between the two studies are due to differences in acoustic bandwidth, energy localization, and coupling efficiency rather than competing resonance behaviors. Combined, these results further suggest that SARS-CoV-2 has frequency-dependent mechanical vulnerability, which may be compromised by broadband, low-intensity ultrasound (for long duration) or by focused, high-frequency ultrasonography (for short duration exposure) when acoustic delivery features are enhanced.

One other study examined viral inactivation by ultrasound at lower frequencies and higher intensities, in combination with chemical sensitizers such as methylene blue (MB). Lu et al. demonstrated with ultrasound in low-mid-MHz (~1.8 MHz) at high acoustic intensities (0.63–2.16 W/cm^2^), especially when combined with MB, 10^5^–10^7^-fold reduction in viral titers, via mainly cavitation-driven and sonochemical pathways involving reactive oxygen species [18]. That study consistently demonstrated that the viral lipid envelope of the enveloped viruses such as Human Parainfluenza Virus Type 3 (HPIV3), influenza, and SARS-CoV-2, not only showed mechanical vulnerability but also chemical vulnerability [19,20]. Nonetheless, such approaches are clearly different from the current work, since they involved chemical intermediates and cavitation, in contrast to our study that deliberately isolated pure acoustic–mechanical effects at high frequency, without the use of sensitizers.

Conversely, a recent low-frequency survey with Ct rises at 40 kHz described using an air-coupled ultrasound setting, in which the transducers were placed about 10 cm away from liquid samples, not including any immersion or acoustic coupling [21]. Physically, an acoustic-based design such as this prohibits a purposeful transfer of ultrasound energy to the liquid phase, especially at frequencies of MHz, due to the extreme attenuation and large acoustic impedance mismatch at the air–liquid interface [22]. Effects, even at kilohertz, would not be considered due to resonance-driven phenomena, but rather to a type of indirect mechanical agitation or experimental artifact. Furthermore, the AFM was the only visualization of viral morphology before ultrasound exposure, offering no direct structural evidence of resonance-induced damage after exposure. These basic methodological limitations preclude comparisons with direct immersion-based, liquid-coupled ultrasound exposure in the current study.

Thus, the current findings indicate that ultrasound–virus interactions strongly depend on frequency, coupling conditions, transducer design, and energy delivery geometry, which have been previously reported in the literature. Here we build on this evidence, with a high-frequency (25 MHz) immersion ultrasound study showing that concentrated high-frequency immersion ultrasound of this nature substantially decreases the viral RNA load, which provides additional support for resonance- or mechanically mediated disruption as a possible approach. Some limitations need to be acknowledged. Viral load reduction was deduced by Ct shifts, not by direct infectivity assays or structural imaging, and this approach did not determine the exact site or the mode of viral damage per se. Second, the experiment was performed in vitro in a distilled water matrix and did not explore the potential impact of biological fluids or host factors. However, the significant effect size and statistical robustness of these findings present a solid basis for future studies.

Future research should be directed toward (i) confirming ultrasound induced viral destruction using high-resolution imaging modalities such as AFM and SEM to study the topography and analyze the surface integrity of virus particles; (ii) investigating whether the mechanical vulnerability observed in this study, focused on the 25 MHz resonance of SARS-CoV-2, can be generalized on other enveloped viruses with different structural dimensions, such as HIV or an influenza virus, or if it requires frequency optimization for each specific viral architecture; (iii) further investigating the use of the method in more controlled extracorporeal conditions, such as blood or plasma circulation systems; (iv) assessing the use of ultrasound in vivo for the inactivation of viruses, following the therapeutic precedents of Lithotripsy [23] for kidney stones and High-Intensity Focused Ultrasound (HIFU) for oncological tissue ablation [24]; and (v) assessing the safety, efficacy, and biological relevance of ultrasound-based viral inactivation techniques in suitable animal models. Together, this work will contribute to a better validation of the results of the different ultrasound platforms and to move ultrasound toward an achievable non-pharmacological antiviral approach.

## 5. Conclusions

The current study demonstrates the potential use of ultrasound waves to significantly reduce viral load in COVID-19-positive samples, as suggested by increased Ct values. Although promising, these results need to be confirmed in further studies and more direct measures of viral structural injury and infectivity. Ultrasound may represent a complement to any arsenal we may already have against pathogenic viruses.

## Figures and Tables

**Table 1:** Distribution of sampled patients according to their demographic data, and the Shapiro–Wilk normality test for the difference between post and pretest Ct values.

Personal Characteristics	No. (44)	Value
Age		
<5	13	29.5
5-	2	4.5
14-	25	56.8
≥75	4	9.1
Mean ± SD ^1^ = 28.67 ± 26.240		
Sex		
Male	18	40.9
Female	26	59.1
Difference between Posttest and Pretest Ct Values		
Min	1	
Max	10	
Mean ± SD ^1^	6.08 ± 2.510	
Shapiro–Wilk Test for Normality		
*p*-value ^2^	0.126	

^1^ SD = Standard Deviation. ^2^ *p* value from the Shapiro–Wilk test for normality (α = 0.05)

**Table 2:** Distribution of changes in viral load categories between pretest and posttest measurements.

Posttest	High Viral Load		Low/Moderate Load		Negative		Total
Pretest	No.	%	No.	%	No.	%	
High Viral Load	2	14.3	12	85.7	0	0	14
Low/Moderate Viral Load	0	0	16	53.3	14	46.7	30
Total	2	4.5	28	63.6	14	31.8	44
Test of Significance	*Monte Carlo p-value: 0.001 (2-sided)*						

**Table 3:** Paired-sample *t*-test results on Ct values of the pretest and posttest samples.

Variable	Pretest	Posttest	Test of Significance	Effect Size (Cohen’s d)
Mean ± SD	28.13 ± 7.090	34.21 ± 5.908	T = −16.065 *p* < 0.001	−2.422

## Data Availability

Research data are available from the corresponding author and will be provided when reasonably requested. Public access to data is limited due to privacy/ethical restrictions, due to involvement of human-driven biological samples.

## Abbreviations

The following abbreviations are used in this manuscript:

AFM	Atomic Force Microscope
COVID-19	Coronavirus Disease 2019
Ct	Cycle Threshold
HIV	Human Immunodeficiency Virus
MHz	Megahertz
MB	Methylene Blue
RT-PCR	Reverse Transcription Polymerase Chain Reaction
SARS-CoV-2	Severe Acute Respiratory Syndrome Coronavirus 2
SEM	Scanning Electron Microscope
US	Ultrasound
VTM	Viral Transport Medium
